# Secondary left ventricular injury with haemopericardium caused by a rib fracture after blunt chest trauma

**DOI:** 10.1186/1749-8090-1-8

**Published:** 2006-03-28

**Authors:** Pankaj Kaul, Ganti Somsekhar, Graeme Macauley

**Affiliations:** 1 Yorkshire Heart Centre, Leeds General Infirmary, Great George Street, Leeds, LS1 3EX, Leeds, UK

## Abstract

Trauma is the third most common cause of death in the West. In the US, approximately 90,000 deaths annually are traumatic in nature and over 75% of casualties from blunt trauma are due to chest injuries. Cardiac injuries from rib fractures following blunt trauma are extremely rare. We report the unusual case of a patient who fell from a height and presented with haemopericardium and haemothorax as a result of left ventricular and lingular lacerations and was sucessfully operated upon.

## Case report

**Figure 1:**
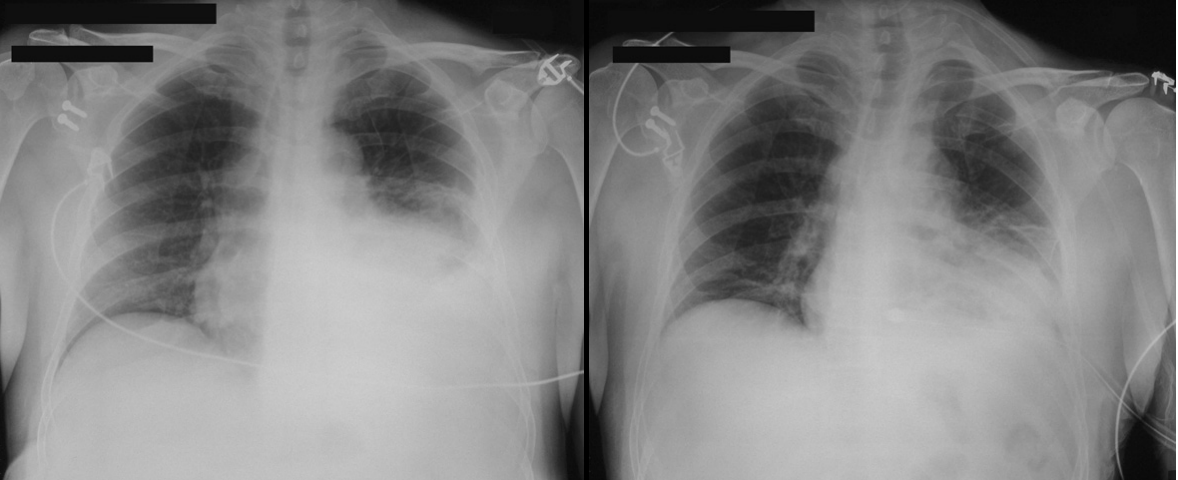
Chest radiograph showing a) left hemothorax and enlarged cardiac silhouette b) residual hemothorax and persistent enlarged cardiac silhouette after pleural tube drainage.

**Figure 2:**
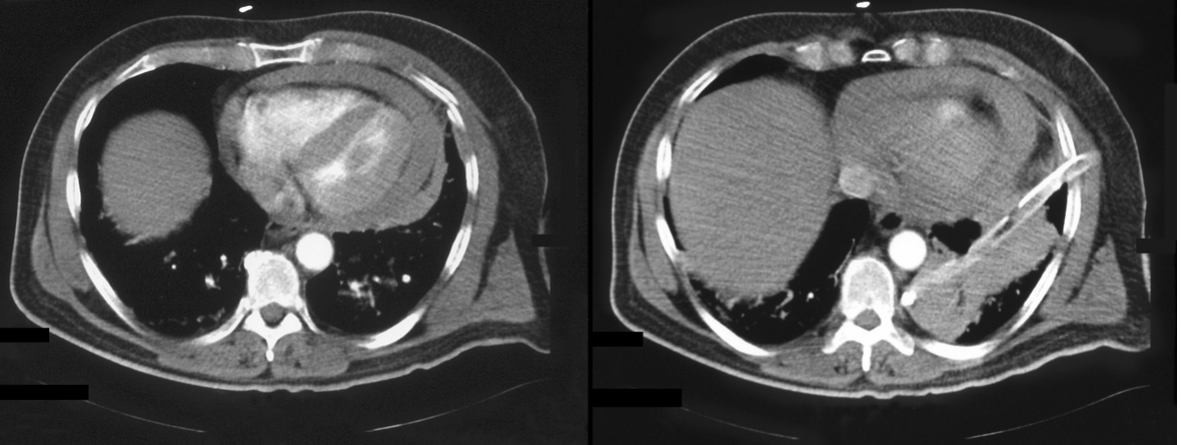
CT Scan of chest showing a) hemopericardium b) hemopericardium and residual left pleural clot and blood despite pleural drainage.

A 55 year old man presented to the accident and emergency department of a district general hospital after having fallen 3 metres from a ladder while cutting his garden hedge. He complained of left sided chest pain and worsening shortness of breath and dizziness. On examination, HR was 155/min, RR 37/min, BP 99/66 mm Hg, and JVP 5 cms above sternal angle. 5^th ^and 6^th ^ribs were tender but there was no obvious crepitus. Breath sounds were diminished in the left base. Chest x-ray revealed fractures of 5^th ^and 6^th ^ribs, a large left hemothorax and enlarged cardiac silhouette (Fig 1a). An intercostal drain was inserted which drained 1 lit of fresh blood but a follow up chest x-ray continued to show a large cardiac shadow (Fig 1b). A CT scan of chest revealed a 2 cm hemopericardium (2a) and residual left pleural blood and clot (Fig 2b). He was transferred to our regional cardiothoracic centre for further management. A transthoracic echocardiogram on arrival confirmed a global pericardial effusion with early tamponade.

Patient was urgently taken to the operation theatre and a median sternotomy performed with bypass standby. The pericardium was tense. It was widely opened and 250 mls of old blood evacuated. There was a 1.5 cm non bleeding superficial left ventricular laceration posterolateral to the apex within a large area of contused left ventricle with a bleeding superficial vein. There was a corresponding 1 cm tear of the pericardium posterior to the phrenic nerve. Left pleura was widely opened and lung inspected. There was a non bleeding laceration of the superior segment of the lingula and about 500 mls of residual clot and blood in the pleural cavity. There were fractures of 5^th ^and 6^th ^ribs with wide displacement of the two segments of the fractured 5^th ^rib. The sharp jagged posterior end of the fractured rib had been displaced medially inside the pleural cavity and had lacerated the lingula and punctured the pericardium and the left ventricle.

The sharp end of the fractured rib was excised, the bleeding ventricular vein was diathermised, the left ventricular contusion and nonbleeding laceration as well as the lingular tear were covered with a generous application of Tissel glue (Baxter biosurgery) and the pleural cavity was evacuated of clot and blood. A posterior pericardial window into left pleural cavity was made. The sternotomy was closed with drainage of left pleural and pericardial cavities.

## Discussion

Cardiac injury from a fractured rib following a blunt trauma to chest from a fall combines the mode and mechanics of penetrating and nonpenetrating injury. 59% of penetrating cardiac injuries are stab wounds, 26% gunshot wounds and 5% others, and 80% of patients die before they reach hospital. Right ventricular injury is most frequent (46%), followed by injuries to left ventricle and right atrium (30% and 11%) [1].

Blunt chest trauma resulting in cardiac injury is not very common. The cardiac injuries can be contusion, heart chamber rupture, ventricular septal rupture, tricuspid valve rupture, coronary arterio venous fistula, mitral tensor apparatus rupture or rarely an aortic cuspal rupture [2,3]. Cardiac contusion is by far the commonest of these, is followed by cardiac rupture uncommonly (0.3%), which, in turn, is associated with mortality as high as 80% [4].

Cardiac injury following a fractured rib due to blunt trauma to chest is an extremely rare event and there are only isolated case reports in world literature [5-10]. All these injuries except one [5] followed motor vehicle accidents [6-10].

Suszoko reported the first cardiac laceration following non-penetrating trauma in 1968 after a man struck his chest while he fell onto a chair [5]. Perchinsky reported one patient who had a cardiac perforation from a rib fragment, 0.007% of all blunt traumas in his series [6].

Glock et al reported two left ventricular perforations with rib fractures [5]. One patient exsanguinated and died and the other one presented with late subacute tamponade and underwent a successful cardiac repair [7]. Bourguignon reported a 60 year old man who had a right ventricular perforation by a rib fragment which came to light following induction of anaesthesia for open fracture of left arm when patient had a rapid cardiovascular collapse. This patient had already had a tube thoracostomy for haemopneumothorax and fractured ribs [8]. Galvin et al described a motor vehicle accident in which a detached fractured rib from a flail chest caused lung perforation and haemopericardium, the full diagnosis of which was appreciated on CT. A thoracotomy resulted in successful salvage [9]. Roth et al described a 33 year old patient with blunt chest trauma and left flail chest. 6 hours after admission patient suddenly drained 1.5 L blood from left chest tube with hypotension and tachycardia. An emergency sternotomy with left anterolateral extension revealed secondary left ventricular perforation by a sharp rib fragment. This was repaired and the outcome was favourable [10].

Although cardiac perforation with rib fracture following blunt chest trauma is rare, it should be kept in mind when 1) there are anterior rib fractures in close proximity to the heart, 2) when there is evidence of raised venous pressure, and 3) when drainage of a haemothorax and replacement of blood losses do not result in expected recovery even though there is no ongoing blood loss. Echocardiogram and CT scan clinch the diagnosis and an early operation can be life saving.
